# Recombinant PTH Infusion in a Child With Sanjad-Sakati Syndrome Refractory to Conventional Therapy

**DOI:** 10.1210/jcemcr/luae059

**Published:** 2024-04-23

**Authors:** Ibrahim Bali, Reem Al Khalifah

**Affiliations:** Division of Pediatric Endocrinology, Department of Pediatrics, College of Medicine, King Saud University, Riyadh, Saudi Arabia, 11421- P. O. Box 800; Pediatric Endocrinology Unit, Department of Pediatrics, King Salman Bin Abdulaziz Medical City, Madina 42319, Saudi Arabia; Division of Pediatric Endocrinology, Department of Pediatrics, College of Medicine, King Saud University, Riyadh, Saudi Arabia, 11421- P. O. Box 800

**Keywords:** infant, newborn, parathyroid hormone, pump, Sanjad-Sakati syndrome, teriparatide

## Abstract

Hypoparathyroidism is the most common endocrinological feature in children with Sanjad-Sakati syndrome. Treatment includes active vitamin D and calcium supplementation. Here, we report a case of a newborn with Sanjad-Sakati syndrome who had severe hypocalcemia since birth who responded to PTH subcutaneous pump infusion. The child was born at 35 weeks with hypocalcemia since the first day of life. The standard medical treatment proved ineffective for the newborn, necessitating the administration of unusually high doses of oral and IV calcium and vitamin D analogue for a 2 months. As a result, intermittent subcutaneous injections of PTH were commenced, resulting in an initial improvement in calcium levels, although this proved to be short-lived. Subsequently, a switch to continuous infusion via a Medtronic Vio pump was made, which unfortunately resulted in iatrogenic hypercalcemia, requiring management of hypercalcemia. Later, calcium carbonate and alfacalcidol were resumed at a lower dosage and continued to have average requirements for patients with hypoparathyroidism. PTH subcutaneous infusion can be highly effective in refractory hypocalcemia cases and can significantly impact the treatment course and facilitate hospital discharge as seen in our case. Careful dosage and monitoring are required to avoid iatrogenic hypercalcemia.

## Introduction

Sanjad-Sakati syndrome (OMIM 241410) or hypoparathyroidism-retardation-dysmorphism syndrome is a rare genetic disorder caused by homozygous or compound heterozygous mutations in the *TBCE* gene (1, 2). Manifestations of growth retardation, congenital hypoparathyroidism, and dysmorphic facial features have been reported in multiple consanguineous families of Arabic descent (1, 3).

Hypoparathyroidism is the most predominant endocrinological feature among children with this syndrome, typically manifesting in early infancy (1, 3). Primary treatment modalities for these children involve the administration of active vitamin D analogs and calcium supplements. Nevertheless, nephrocalcinosis and renal stones caused by hypercalciuria remain a major challenge in the treatment of hypoparathyroidism, especially in patients with this syndrome (4‐6). Therefore, close monitoring and adjustment of therapeutic doses are required to prevent complications.

## Case Presentation

We report the case of a newborn with Sanjad-Sakati syndrome who had severe refractory hypocalcemia and was unresponsive to high doses of vitamin D analogs and calcium supplements over a 2-month period. Consequently, the initiation of PTH subcutaneous infusion through a pump was necessitated for management.

The patient was a female delivered via cesarean section at 35 weeks of gestation because of nonreassuring cardiotocography and the presence of a thick meconium. Her Apgar scores were 4 and 8, respectively. Her birth weight was 1.6 kg (third percentile), length measured 42 cm (third percentile), and head circumference was 30 cm (tenth percentile). Owing to prematurity, intrauterine growth retardation, and facial dysmorphism, she was promptly admitted to the neonatal intensive care unit. Initial patient assessment at the neonatal intensive care unit revealed the following characteristics: a narrow face, deep-seated eyes, micrognathia, and small hands and feet. The genetic team examined her, suspecting Sanjad-Sakati syndrome (Middle East syndrome) based on her clinical features.

## Diagnostic Assessment

On the second day after birth, she developed hypocalcemia with a calcium level of 1.8 mmol/L (7.2 mg/dL) (normal reference range, 2-2.6 mmol/L; 8.42-10.4 mg/dL), phosphorus level of 2.58 mmol/L (7.9 mg/dL) (normal reference range, 1.45-3 mmol/L; 4.3-9.3 mg/dL), magnesium level of 0.54 mmol/L (1.3 mg/dL) (normal reference range, 0.85-1.10 mmol/L; 1.7-2.2 mg/dL), a PTH level of 0.4 pmol/L (normal reference range, 1.6-6.9 pmol/L), and a 25-hydroxy vitamin D concentration of 48.4 nmol/L (normal reference range, 50-250 nmol/L) (Table 1).

**Table 1.** Changes in calcium level and medications intake over the course of admission

Patient's age	Total calcium, in mmol/L (Ref. 2.2-2.7 mmol/L, 8.8-10.8 mg/dL)	Phosphorus, in mmol/L (Ref. 1.45-2.1 mmol/L, 4.5-6.5 mg/dL)	Total calcium intake mg/kg/day	Cholecalciferol, IU/day	Alfacalcidol, mcg/kg/day	Teriparatide, mcg/kg/day
Day 2	1.8 mmol/L (7.2 mg/dL)	2.58 mmol/L (7.9 mg/dL)	50 mg/kg/day	—	—	—
Day 6	1.54 mmol/L (6.2 mg/dL)	2.5 mmol/L (7.8 mg/dL)	90 mg/kg/day	1000 IU/day	1 mcg/kg/day	—
Day 21	1.58 mmol/L (6.3 mg/dL)	2.5 mmol/L (7.8 mg/dL)	340 mg/kg/day	1000 IU/day	1 mcg/kg/day	—
Day 60*^a^*	2 mmol/L (8 mg/dL)	2.4 mmol/L (7.4 mg/dL)	600 mg/kg/day	800 IU/day	2 mcg/kg/day	3 mcg/kg/day (injections)
Day 66*^a^*	2.1 mmol/L (8.4 mg/dL)	2.5 mmol/L (7.8 mg/dL)	600 mg/kg/day	800 IU/day	2 mcg/kg/day	3 mcg/kg/day (pump)
Day 67*^b^*	3.9 mmol/L (15.6 mg/dL)	2.1 mmol/L (6.5 mg/dL)	—	—	—	—
Day 76*^c^*	2 mmol/L (8 mg/dL)	2.3 mmol/L (7.1 mg/dL)	220 mg/kg/day	800 IU/day	1 mcg/kg/day	—
18 mo	2.14 mmol/L (8.6 mg/dL)	1.7 mmol/L (5.3 mg/dL)	40 mg/kg/day	400 IU/day	0.3 mcg/kg/day	—
3 y	2.15 mmol/L (8.6 mg/dL)	1.9 mmol/L (5.9 mg/dL)	60 mg/kg/day	400 IU/day	0.2 mcg/kg/day	—
4 years	2.27 mmol/L (9 mg/dL)	1.9 mmol/L (5.9 mg/dL)	103 mg/kg/day	400 IU/day	0.2 mcg/kg/day	—
6 years	2.1 mmol/L (8.4 mg/dL)	1.96 mmol/L (6 mg/dL)	200 mg/kg/day	—	0.1 mcg/kg/day	—

^*a*^Weight 2 kg.

^*b*^Treated for iatrogenic hypercalcemia.

^*c*^Discharged home.

## Treatment

She was diagnosed with hypoparathyroidism and started on alfacalcidol at 0.125 mcg/kg/daily, elemental calcium carbonate at 50 mg/kg/day, and magnesium replacement for a brief period. Despite initial therapeutic efforts, maintaining her calcium levels proved challenging; therefore, her doses were increased as follows: alfacalcidol of 1.17 mcg/kg/daily and elemental calcium carbonate of 180 mg/kg/day with a continuous calcium infusion of 2 mg/kg/h. The total calcium intake was approximately 600 mg/kg/day until the age of 2 months.

Trials of IV alfacalcidol and oral calcitriol failed to decrease the calcium requirements; furthermore, attempts to enhance calcium absorption through overnight continuous breastfeeding failed to eliminate the need for IV calcium. During this period, her urine calcium-to-creatinine ratio was 6.8, renal ultrasound showed normal kidney echogenicity bilaterally, and there was no vomiting or diarrhea that could have impacted her calcium absorption. Therefore, the team decided to initiate a trial of subcutaneous PTH treatment (teriparatide 250 mcg/mL) 1 mcg/kg/day to facilitate the discontinuation of IV calcium. However, because of challenges in IV access, the patient developed calcium extravasation resulting in subcutaneous calcification and skin necrosis at the right wrist and left ankle. Despite this complication, PTH injections successfully increased the blood calcium levels, although the sustainability was limited. Therefore, the dosage was gradually increased to 3 mcg/day (1.5 mcg/kg/day) twice daily, resulting in the amelioration of hypocalcemia and hyperphosphatemia. Eventually, she was successfully weaned off of calcium infusion, but remained on high doses of alfacalcidol and oral calcium.

At 66 days of age, the patient was transitioned from intermittent PTH injections administered twice daily to continuous subcutaneous PTH infusion through a Medtronic MiniMed Vio pump at a dose of 0.125 mcg/h (infused at a rate of 0.025 units/h), resulting in a total dosage of 3 mcg in 24 hours. Concurrently, high doses of alfacalcidol and oral calcium carbonate were administered. The Medtronic MiniMed Vio infusion set 6Ml/60Cm was inserted in the right thigh to infuse PTH. Monitoring the calcium level every 6 hours was challenging because of difficult IV access. Therefore, calcium levels were checked at night, and the results were not relayed to the on-call team. Twenty-four hours after initiating PTH infusion, the patient developed symptomatic hypercalcemia with a calcium level reaching 3.9 mmol/L (15.6 mg/dL). Consequently, all medications were discontinued, and she was started on hypercalcemia management, including fluid hydration, furosemide 1 mg/kg twice per day, and hydrocortisone 1 mg/kg every 6 hours for 48 hours. Calcium carbonate and alfacalcidol were resumed 72 hours after stopping all treatments, and the calcium levels reached the low normal range.

## Outcome and Follow-up

**Figure 1.**
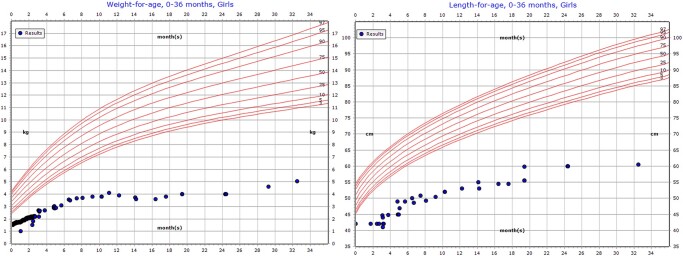
Growth parameters in the first 3 years of life.

Since then, the patient has remained stable only on average oral calcium and alfacalcidol doses. She was discharged home after 3 days on elemental calcium of 110 mg every 6 and 1 mcg of alfacalcidol. At the age of 3 months, her blood vitamin D concentration was 130 nmol/L. Occasionally, over the following years, her calcium treatment was increased during episodes of viral infection, but those calcium needs were typical for her disease. Figure 1 shows the patient's growth parameters over the first 36 months after birth. However, the growth parameters are far below the plotting area of the growth curve and cannot be plotted after the age of 3 years.

At the last follow up at 6 years of age, her weight was 5.80 kg (SD −23.05), and her height was 69.00 cm (SD −13.11). Her calcium level was maintained at 200 mg/kg/day together with 0.1 mcg of alfacalcidol. The last renal ultrasound performed at 5 years of age showed normal bilateral kidney echogenicity and preserved cortical thickness.

## Discussion

We report a case of Sanjad-Sakati syndrome presenting with unusually severe refractory hypocalcemia during the early newborn period. The standard medical treatment proved ineffective for the newborn, necessitating the administration of unusually high doses of oral and IV calcium and vitamin D analog for 2 months. To the best of our knowledge, PTH replacement therapy through pump infusion has not been previously used for the management of refractory severe hypocalcemia in newborns with hypoparathyroidism. Although concerns regarding long-term exposure to teriparatide and the risk of osteosarcoma still exist, a balance between the ongoing treatment challenges from difficult IV access, high-dose calcium infusion, and possible complications from calcium extravasation needs to be considered.

The short-term use of continuous subcutaneous PTH through pump infusion overcame the resistance to treatment and led to a decrease in the calcium and alfacalcidol requirements to the typical doses used in children with hypoparathyroidism.

Typically, conventional therapy for hypoparathyroidism with calcium and active vitamin D analogs is sufficient to control calcium levels in most cases. However, in some patients, hypoparathyroidism is difficult to control, even at high doses. In addition, prolonged use of high doses of conventional treatment can increase the risk of complications such as hypercalciuria, nephrocalcinosis, and ectopic soft tissue calcification (4, 7).

Refractory hypocalcemia in patients with hypoparathyroidism managed with conventional therapy can result in decreased response of the physiological effects of the parathyroid hormone on the kidneys, leading to hypercalciuria, which limits the effect of calcium administered orally or parenterally (4). In addition, calcium originating from bone resorption is influenced by both PTH and calcitriol; therefore, in the absence of PTH, the amount of calcium originating from bone resorption can be affected even if active vitamin D is administered and may even contribute to refractory hypocalcemia in some patients with hypoparathyroidism (7).

PTH therapy can be a therapeutic option for infants by replacing the physiological role of the missing hormone in maintaining normal calcium levels and limiting possible complications related to uncontrolled hypercalciuria (7). Teriparatide (PTH 1-34) is the most widely available form of PTH and has been used in the management of children with different etiologies of hypoparathyroidism as a daily or twice daily subcutaneous injection (8, 9). Studies have indicated no difference compared with calcitriol in maintaining mean urine calcium levels within the normal range. This lack of additional effects may be related to the short half-life of PTH 1-34 (10). In addition, a newer form of recombinant human PTH with longer half-lives (1-84) has been approved by the US Food and Drug Administration for the management of chronic hypoparathyroidism in adults (7, 11). It has also been used to treat children with hypoparathyroidism, with promising therapeutic effects (12).

Teriparatide was administered during the newborn period in 2 previous cases. The first child was a 17-day-old boy who presented with hypoparathyroidism leading to profound hypocalcemia and requiring a total calcium intake of 105 mg/kg/day (13). The calcium level normalized with the addition of teriparatide 4 mcg subcutaneous injection. However, we were unaware of whether the injections were continued for a substantial period. The second infant was treated with teriparatide twice daily following subcutaneous injections. The infant was born with intra-uterine growth restriction at 31 weeks of age, with Sanjad-Sakati syndrome and recurrent episodes of severe hypocalcemia (9). Teriparatide was administered at 2 weeks corrected gestational age, at a dose of 1 mcg/kg/dose and continued for 12 days only.

Unlike our case, the 2 previous cases in the literature responded well to intermittent injections. This may be due to the early initiation of PTH replacement therapy. Although the same total PTH dose was administered daily, this resulted in unexpected hypercalcemia. This further supports previous reports of lower PTH needs with continuous administration (14). However, it remains unclear why the calcium requirement and vitamin D analog levels decreased by more than 50% after the child recovered from iatrogenic hypercalcemia caused by teriparatide and remained unchanged for the following 6 years.

Winer et al (14). compared the use of twice-daily teriparatide injections for pump delivery in a randomized crossover study in children aged 7 to 20 years with severe hypoparathyroidism from different causes. The use of pump delivery was more physiologic and associated with minimal fluctuations in both serum and urine calcium compared to a biphasic pattern related to twice-daily injections. In addition, pump delivery was associated with a 62% reduction in the daily PTH 1-34 dose, which may explain the hypercalcemia that occurred in our patient when switching from subcutaneous injections to pump delivery. The typical required dose among pump users was 0.32 ± 0.04 μ/kg/d compared with 0.85 ± 0.11 μ/kg/d with twice-daily injections.

Another study used continuous subcutaneous PTH infusion in the treatment of 3 children with severe hypoparathyroidism with a starting dose of 1 to 2.6 mcg/kg/day followed by a reduction to a maintenance dose of 0.1 to 0.5 mcg/kg/day directed by serial calcium monitoring, which successfully achieved normal serum and urine calcium levels over a period of 3 years (15). They used a lower starting dose than that used in our patient, and the maintenance doses were far less than those of the subcutaneous PTH 1-34 used in the reported cases.

## Learning Points

- Newborns with Sanjad-Sakati syndrome can present with severe refractory hypocalcemia unresponsive to high doses of vitamin D analogs and calcium supplements.
- The early initiation of teriparatide for few days for refractory hypocalcemia cases can significantly impact the treatment course and facilitate early hospital discharge, as seen in our case.
- Future studies are required to explore the dosing, effectiveness, and safety of Teriparatide treatment in infants diagnosed with Sanjad-Sakati syndrome or other types of hypoparathyroidism.

## Data Availability

Data sharing is not applicable to this article as no datasets were generated or analyzed during the current study.
